# Spectral Correlation Demodulation Analysis for Fault Diagnosis of Planetary Gearboxes

**DOI:** 10.3390/s25092694

**Published:** 2025-04-24

**Authors:** Xiaohui Duan, Rongzhou Lin, Zhipeng Feng

**Affiliations:** School of Mechanical Engineering, University of Science and Technology Beijing, Beijing 100083, China; duanxiaohui@xs.ustb.edu.cn (X.D.); d202110307@xs.ustb.edu.cn (R.L.)

**Keywords:** planetary gearbox, fault diagnosis, demodulation analysis, spectral correlation

## Abstract

Planetary gearbox vibrations exhibit complex amplitude modulation (AM) and frequency modulation (FM) characteristics. The spectral correlation (SC) can reveal the cyclostationarity of rotating machinery signals, but previous studies have modeled gearbox signals as impulse response signals rather than AM–FM signals. This paper presents a fault diagnosis method for planetary gearboxes via SC based on the AM–FM model. Firstly, the theoretical expression for the spectral correlation characteristics of AM-FM signals is derived, showing that their demodulation features consist of discrete and grouped points rather than continuous vertical lines, and demonstrating the capability of SC to reveal the cyclostationarity of AM–FM signals. Then, the theoretical SC characteristics of planetary gearbox vibration signals under gear localized fault conditions are derived in closed form, providing theoretical guidance for fault diagnosis. The theoretical derivations are validated experimentally, and the localized faults on the ring, sun, and planet gears are successfully diagnosed.

## 1. Introduction

Planetary gearboxes are widely used in fields such as wind power, aerospace, and heavy-duty machinery due to their high power density and compact structure. However, the harsh operating conditions lead to a high failure rate. Once a failure occurs, it can result in equipment downtime or even safety incidents. Therefore, developing efficient fault diagnosis methods is crucial for ensuring safe operation and reducing maintenance costs [1,2].

Planetary gearbox vibration signals involve multi-source and time-varying excitations, which result in complex modulation characteristics and difficulty in fault diagnosis [3]. A planetary gearbox often consists of a sun gear, planet gears, a ring gear, and a planet carrier, with the ring gear typically fixed and the planet gears meshing with both the sun gear and the ring gear. When faults such as root cracks or surface spallings occur in the gear, the meshing stiffness of the faulty gear drops abnormally during engagement, leading to periodic modulation components in the vibration signal [4]. Typically, vibration sensors are installed at fixed positions on the gearbox surface, while the position of the meshing points changes over time due to the revolution of the planet gears, making the transmission path of the vibration signal from the meshing point to the sensor time-varying, which results in an amplitude modulation effect on the fault signal. To guide the fault diagnosis of planetary gearboxes, researchers have established vibration signal models for both normal and faulty states and derived the theoretical spectral structure of their Fourier spectra [5]. However, the frequency components in real-world planetary gearbox signals are densely distributed, making fault feature extraction to remain challenging in practical applications [6,7].

Cyclostationarity is an intrinsic characteristic of most rotating machinery signals. For example, the rotating vibrations of shafts, the meshing vibrations of gears, and the reciprocating vibrations of engines exhibit specific periodic repetitions, classifying these signals as cyclostationary signals. Spectral correlation (SC) is a method for detecting the cyclostationarity of signals, and it is a function of spectral frequency and cyclic frequency [8,9,10]. Various fast algorithms for SC have been proposed, such as Averaged Cyclic Periodogram, the Cyclic Modulation Spectrum, and the Fast-SC, facilitating its engineering applications [11,12]. It has been proved that the result of integrating SC along the spectral frequency is equivalent to the squared envelope spectrum, showing that SC can be viewed as an expansion of the squared envelope spectrum in the spectral frequency dimension [13]. Therefore, SC can be applied as a demodulation analysis method to extract the hidden modulation features of cyclostationary signals. A traditional demodulation analysis method based on envelope signals can directly reveal modulation component features, but it cannot pinpoint the carrier source of modulation features. For example, sidebands may originate from multiple frequency bands, making it difficult to determine the accurate meshing order of modulation features in the envelope spectrum. The SC addresses this issue by displaying modulation features in both the cyclic and spectral frequency axes, which correspond to the carrier band. Thus, the SC method offers more comprehensive demodulation information.

Currently, research on fault diagnosis based on SC primarily focuses on bearing diagnostics [14]. The bearing fault signals are usually modeled as periodic impulse responses. The SC represents them as vertical lines perpendicular to the cyclic frequency axis in the bivariate spectrum, with spectral and cyclic frequencies corresponding to the resonant frequency band and fault characteristic frequency of the bearing, respectively [15]. The improved envelope spectrum, obtained by integrating the spectral frequencies of interest based on spectral coherence (SCoh), can enhance the bearing fault features [16,17]. Nevertheless, due to the unknown resonance frequencies, it is still necessary to manually locate the characteristic frequency bands in a two-dimensional plane. The improved envelope spectrum via feature optimization gram has been proposed to automatically extract the optimal analysis frequency band [18,19]. It utilizes a 1/3 binary tree to partition the Nyquist frequency and constructs diagnostic indicators based on the IES of each frequency band. The SC method allows for a more straightforward identification of the frequency bands associated with bearing fault features.

The vibration signals of planetary gearboxes exhibit various modulation phenomena, classifying them as typical second-order cyclostationary signals. Researchers have applied SC to the fault diagnosis for planetary gearboxes, while the reported works modeled gear fault signals as periodic impulse responses rather than AM–FM signals [20,21]. This results in the need to search for resonance frequency bands in the entire Nyquist frequency, similar to bearing fault diagnosis, while overlooking the gear meshing frequency as a modulation carrier, thus complicating gear fault diagnosis [21]. To facilitate the gear diagnosis based on SC, clarifying the demodulation characteristics of AM–FM signals is essential. However, the theoretical SC patterns of the AM–FM signal model have yet to be systematically summarized, hindering the application of the SC method in gearbox diagnosis.

This paper proposes to diagnose planetary gearbox faults using an SC-based method. Firstly, based on the principle of SC demodulation in the frequency domain, the theoretical demodulation characteristics of the AM–FM signal in the bivariate spectrum of SC are deduced. Further, the feature distributions of planetary gearboxes under gear fault states in SC are derived in closed form, which provide theoretical guidance to the fault diagnosis of planetary gearboxes based on SC. Finally, the effectiveness of the proposed theory is demonstrated through the experimental study on fault diagnosis of a laboratory planetary gearbox based on SC.

## 2. Spectral Correlation

In general, cyclostationary signals are characterized by periodic variations in their statistical properties. An n-th order cyclostationary (CSn) signal means its n-th moment is periodic. For instance, the first-order statistical moment of CS1 signals is periodic:

(1) $$C_{x}\left(t\right)=E\left[x\left(t\right)\right]=C_{x}\left(t+T\right),$$

where E[·] indicates the ensemble average operator, and *T* is for the cyclo-period, corresponding to the cyclical frequency *α* = 1/*T*. In rotating machinery, the rotating vibrations of rotors/shafts and their harmonics are CS1 signals, and they exhibit periodic changes with phase, remaining stable over multiple cycles. The second-order statistical moment of CS2 signals, also known as the instantaneous autocorrelation function, is a periodic function:

(2) $$R_{x}\left(t,\tau \right)=E\left[x\left(t\right)x{\left(t-\tau \right)}^{*}\right]=R_{x}\left(t+T,\tau \right),$$

where *t* is the time-lag variable, and ∗ means the complex conjugate. The periodicity of the autocorrelation function indicates that signals with a specific oscillation pattern that reoccur with a stable period can be classified as cyclostationary; thereby, the SC2 encompasses a wide variety of signal forms. The periodic impulsive response signals of faulty bearings and the periodic modulation signals of gear meshing are CS2 signals.

After time-series discretization, $R_{x}\left(t,\tau \right)$ becomes the following

(3) $$R_{x}\left(t_{n},\tau \right)=\underset{N\to \infty }{\lim}\frac{1}{\left(2N+1\right)}\sum_{n=-N}^{N}\left[x\left(n/F_{s}\right)x{\left(n/F_{s}-\tau \right)}^{*}\right]$$

where *F*_s_ is the sampling frequency, the considered time range is [−*N*/*F*_s_, *N*/*F*_s_], and the time series is *t_n_* = *n*/*F*_s_, *n*∈ [−*N*, …, −1, 0, 1, …, *N*]. Furthermore, the SC is the double discrete Fourier transform of Equation (2):

(4) $$\mathrm{SC}(\alpha ,f)=\;\;\underset{N\to \infty }{\lim}\frac{1}{(2N+1)F_{s}}\sum_{n=-N}^{N}\sum_{m=-\infty }^{\infty }R_{x}\left(t_{n},\tau_{m}\right)e^{-j2\pi n\frac{\alpha }{F_{s}}}e^{-j2\pi m\frac{f}{F_{s}}},\tau_{m}=m/F_{s}$$

where α denotes the cyclic frequency, and *f* denotes the spectral frequency. SC generates a two-dimensional representation composed of two frequencies: the spectral frequency has the same meaning as in the Fourier spectrum, while the cyclic frequency corresponds to the cyclic period *T* = 1/*α* of the signal. Another equivalent definition of SC in the frequency domain is as follows

(5) $$\begin{array}{c} \mathrm{SC}(\alpha ,f)\;=\underset{N\to \infty }{\lim}\frac{1}{W}E\left\{F_{W}{\left[x\left(t\right)\right]}^{*}F_{W}\left[x\left(t\right)e^{-j2\pi \alpha t}\right]\right\}\\ \;\;=\underset{N\to \infty }{\lim}\frac{1}{W}E\left\{X_{W}^{*}(f)X_{W}(f+\alpha )\right\}, \end{array}$$

where $F_{W}\left[x\left(t\right)\right]=X_{W}(f)$ is the Fourier transform of signal *x*(*t*) during time *W*. Equation (5) interprets the SC as the ensemble average of the frequency domain product in frequency *f* and *f* + *a* of the segmented signals. The value of SC is expressed in terms of energy density, as it belongs to a quadratic distribution. Specifically, when *α* = 0, the SC becomes the power spectral density. The non-zero value in the vertical slice ${\mathrm{SC}}^{\alpha }\;(f)$ (a function of *f*) indicates the presence of modulation features with frequency α at the carrier frequency *f*. Thus, SC can be considered as a demodulation spectrum that displays information along the carrier frequency axis.

The amplitude of the measured signal’s SC often distributes uneven, with significantly high values in frequency bands associated with resonance, meshing, or colored noise, while being very low in others. Thus, obtaining the SCoh by normalizing the signal power is a commonly adopted approach:

(6) $$\mathrm{SCoh}(\alpha ,f)=\frac{\mathrm{SC}(\alpha ,f)}{\sqrt{\mathrm{SC}(0,f)\mathrm{SC}(0,f+\alpha )}}$$

The amplitude of SCoh ranges between 0 and 1, representing the relative modulation strength of the signal within local frequency bands. The SCoh narrows uneven distributions across different frequency bands in the CS, thereby highlighting modulation features that were previously obscured due to low amplitude.

## 3. Demodulation Analysis via Spectral Correlation

The fault signals are usually modeled as an AM–FM signal in gear fault diagnosis. The AM–FM signal can be considered as a sum of the fundamental frequency and its harmonics through Fourier series, so that the spectrum is composed of discrete peaks. For a stationary signal, the frequency domain characteristics extracted at different times are fundamentally the same. Therefore, we can temporarily disregard the ensemble averaging operation and derive theoretical SC characteristics using the Fourier transform of the AM–FM signal.

**Figure 1:**
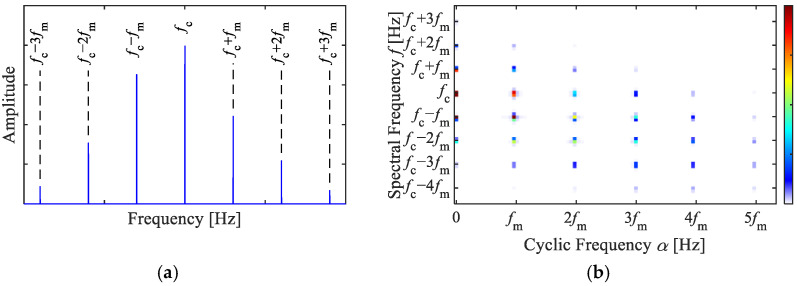
AM–FM signal, (**a**) Fourier spectrum, (**b**) SC.

Consider an AM–FM signal as follows:

(7) $$x(t)=\left[1-A\cos\left(2\pi f_{m}t+\phi \right)\right]\cos\left[2\pi f_{c}t+B\sin\left(2\pi f_{m}t+\varphi \right)+\theta \right],$$

where *f*_m_ is the modulation frequency, *f*_c_ is the carrier frequency, *A* is the intensity coefficient for amplitude modulation, *B* is the intensity coefficient for frequency modulation, and ϕ, φ, and θ are the initial phases in AM, FM, and the carrier components, and they are random values in [0, 2π]. Its Fourier transform is as follows:

(8) $$X(f)=\sum_{m=-\infty }^{\infty }\delta \left[f-\left(f_{c}+mf_{m}\right)\right]\exp[j(m\varphi +\theta )]\left[J_{m}(B)+\frac{A}{2}J_{m+1}(B)\exp(j\phi )+\frac{A}{2}J_{m-1}(B)\exp(-j\phi )\right]$$

where $J_{m}(B)$ is the Bessel function of the first kind with argument z at order m, and δ(f) is the Dirac Delta function. From Equation (8), the Fourier spectrum of an AM–FM signal exhibits peaks at $f_{c}\;\pm mf_{m}$, while the values at all other positions are zero, as shown in Figure 1a.

According to Equation (5), the SC(α,f) of the AM–FM signal is as follows

(9) $$\mathrm{SC}(\alpha ,f)\;=\left\{\begin{array}{l} \underset{N\to \infty }{\lim}\frac{1}{W}E\left\{X_{W}{\left(f\right)}^{*}X_{W}(f+\alpha )\right\}\;\mathrm{if}\;\alpha =m^{\prime }f_{m}\;\\ 0\;\mathrm{other}\;\alpha . \end{array}\right.$$

where SC(α,f) exhibits peaks only when both X(f) and X(f+α) are non-zero, otherwise, SC(α,f)= 0. When α=0, SC(0,f) represents the squared envelope spectrum. When $\alpha =f_{m}$, the $\mathrm{SC}\;(f_{m},f)$ exhibits peaks at $f\;=\;\;f_{c}\;\;\pm \;mf_{m}$ through the product of $X(f_{c}\;\pm mf_{m})$ and $X(\;f_{c}\;\pm mf_{m}+f_{m}\;)$. Similarly, when $\alpha =m^{\prime }f_{m}$, (*m* = 1, 2, 3…), then $\mathrm{SC}\;(m^{\prime }f_{m},f)$ exhibits peaks at $f\;=\;\;f_{c}\;\;\pm \;mf_{m}$ through the product of $X(f_{c}\;\pm mf_{m})$ and $X(\;f_{c}\;\pm mf_{m}+m^{\prime }f_{m}\;)$. At other values of *f* and α, SC(α,f)=0. The obtained SC of an AM–FM signal is shown in Figure 1b, showing that the characteristic peaks appear as a series of discrete points.

Based on the above, SC can detect the pairwise spacing of X(f) and X(f+α) within the Fourier spectrum, which displays the frequency difference α and the smaller frequency *f* on the axes of cyclic and spectral frequency in a two-dimensional spectrum. By applying SC for the demodulation analysis of AM–FM signals, the discrete points in the two-dimensional spectrum can indicate the AM and FM components in the signal. Their modulation frequency and carrier frequency can be recognized, respectively, by their cyclic frequencies and spectral frequencies, which are similar to the generalized correlation characteristics of impulse signals. However, impulse signals manifest as vertical lines, providing an approximate range about the carrier. In contrast, AM–FM signals appear as discrete clusters of points, with their spectral frequencies equal the sum or difference of the carrier frequency and the modulation frequency, offering more accurate information for locating the modulation source. Therefore, the SC method is an effective tool for the demodulation analysis of signals with complex AM and FM characteristics.

## 4. Planetary Gearbox Fault Characteristics in Spectral Correlation

The vibration signals of the planetary gearbox are modulated by factors such as localized faults and transmission paths, rendering them cyclostationary and can be demodulated by the SC method. To diagnose the fault of planetary gearboxes based on SC, it is essential to clarify the theoretical SC characteristics of the fault signals. This section will derive the spectral correlation expression based on the fault signal model of planetary gearboxes. Due to differences in modulation patterns of different gear fault modes, the SC representation under various cases will be discussed separately.

### 4.1. Ring Gear Fault Case

In the case of ring gear faults, the periodic meshing of the faulty tooth causes an AM–FM effect on the meshing frequency. Since the transmission path between the sensor and the housing surface is fixed, no additional modulation effects need to be considered [5]. Thus, the signal of ring gear fault can be expressed as a typical AM–FM signal:

(10) $$x_{r}(t)=\left[1-A\cos\left(2\pi f_{r}t+\phi \right)\right]\cdot \cos\left[2\pi f_{m}t+B\sin\left(2\pi f_{r}t+\varphi \right)+\theta \right],$$

where $f_{r}$ is the fault characteristic frequency of the ring gear, and *f*_m_ is the meshing frequency. Its Fourier spectrum is as follows

(11) $$X_{r}(f)=\sum_{m=-\infty }^{\infty }\delta \left[f-\left(f_{m}+mf_{r}\right)\right]\exp[j(m\varphi +\theta )]\left[J_{m}(B)+\frac{A}{2}J_{m+1}(B)\exp(j\phi )+\frac{A}{2}J_{m-1}(B)\exp(-j\phi )\right]$$

Equation (8) shows that the Fourier spectrum presents peaks at $f_{m}\;\pm mf_{r}$ and is zero at all other frequencies. The corresponding ${\mathrm{SC}}_{r}(\alpha ,f)$ of $x_{r}(t)$ can be derived as follows

(12) $${\mathrm{SC}}_{r}(\alpha ,f)\;=\left\{\begin{array}{ll} \underset{N\to \infty }{\lim}\frac{1}{W}E\left\{X_{r-W}{\left(f\right)}^{*}X_{r-W}(f+\alpha )\right\} & \mathrm{if}\;\alpha =m^{\prime }f_{r}\\ 0 & \mathrm{other}\;\alpha . \end{array}\right.$$

Verbally, the SC of the ring gear fault signal exhibits peaks at SC_r_($m^{\prime }f_{r}$, $f_{m}\;\pm \;mf_{r}$), where *m* and *m*′ are natural numbers, and they display equal horizontal and vertical spacing in the bivariate map, similar to Figure 1b.

### 4.2. Sun Gear Fault Case

In the case of sun gear faults, the fault sun gear induces simultaneous AM and FM on the gear meshing frequency, and the time-varying transmission path introduces amplitude modulation by the sun gear rotating frequency on the vibration signal, which can be characterized by a Hanning window [5]. Thus, the signal of a localized sun gear fault can be expressed as follows:

(13) $$x_{s}(t)=\left[1-\cos\left(2\pi f_{s}^{\left(r\right)}t\right)\right]\cdot \left[1-A\cos\left(2\pi f_{s}t+\phi \right)\right]\;\;\cdot \cos\left[2\pi f_{m}t+B\sin\left(2\pi f_{s}t+\varphi \right)+\theta \right]$$

where $f_{m}$ is the meshing frequency, $f_{s}$ is the fault characteristic frequency of the sun gear, and $f_{s}^{\left(r\right)}$ is its rotating frequency. The Fourier spectrum of $x_{s}(t)$ can be derived as follows:

(14) $$\left\{\begin{array}{c} X_{s}(f)=P_{s1}(f)+P_{s2}(f)+P_{s3}(f)\\ P_{s1}(f)=\sum_{m=-\infty }^{\infty }\delta \left[f-\left(f_{m}+mf_{s}\right)\right]\exp[j(m\varphi +\theta )]\left[J_{m}(B)+\frac{A}{2}J_{m-1}(B)\exp(j\phi )+\frac{A}{2}J_{m+1}(B)\exp(-j\phi )\right]\\ P_{s2}(f)=-\frac{1}{2}\sum_{m=-\infty }^{\infty }\delta \left[f-\left(f_{m}+mf_{s}+f_{s}^{\left(r\right)}\right)\right]\exp[j(m\varphi +\theta )]\left[J_{m}(B)+\frac{A}{2}J_{m-1}(B)\exp(j\phi )+\frac{A}{2}J_{m+1}(B)\exp(-j\phi )\right]\\ P_{s3}(f)=-\frac{1}{2}\sum_{m=-\infty }^{\infty }\delta \left[f-\left(f_{m}+mf_{s}-f_{s}^{\left(r\right)}\right)\right]\exp[j(m\varphi +\theta )]\left[J_{m}(B)+\frac{A}{2}J_{m-1}(B)\exp(j\phi )+\frac{A}{2}J_{m+1}(B)\exp(-j\phi )\right]. \end{array}\right.$$

Based on the locations of spectral peaks, it can be simplistically expressed as follows:

(15) $$X_{s}(f)=\left\{\begin{array}{ll} P_{s1}(f) & \mathrm{if}\;f=f_{m}+mf_{s}\\ P_{s2}(f) & \mathrm{if}\;f=f_{m}+mf_{s}+f_{s}^{\left(r\right)}\\ P_{s3}(f) & \mathrm{if}\;f=f_{m}+mf_{s}-f_{s}^{\left(r\right)}\\ 0 & \mathrm{other}\;f. \end{array}\right.$$

Equation (15) indicates that the peaks of the sun gear fault signal occur at frequencies $f_{m}\;\pm mf_{s}$ and $f_{m}\;\pm \;mf_{s}\;\pm \;f_{s}^{\left(\;r\;\right)}$. The ${\mathrm{SC}}_{s}(\alpha ,f)$ of $x_{s}(t)$ can be expressed as follows:

(16) $${\mathrm{SC}}_{s}(\alpha ,f)\;=\left\{\begin{array}{ll} \underset{N\to \infty }{\lim}\frac{1}{W}E\left\{P_{s1-W}{\left(f\right)}^{*}P_{s1-W}(f+\alpha )+P_{s2-W}{\left(f\right)}^{*}P_{s2-W}(f+\alpha )+P_{s3-W}{\left(f\right)}^{*}P_{s3-W}(f+\alpha )\right\} & \mathrm{if}\;\alpha =m^{\prime }f_{s}\\ \underset{N\to \infty }{\lim}\frac{1}{W}E\left\{P_{s1-W}{\left(f\right)}^{*}P_{s2-W}(f+\alpha )+P_{s3-W}{\left(f\right)}^{*}P_{s1-W}(f+\alpha )\right\} & \mathrm{if}\;\alpha =m^{\prime }f_{s}\;+\;f_{s}^{\left(r\right)}\\ \underset{N\to \infty }{\lim}\frac{1}{W}E\left\{P_{s1-W}{\left(f\right)}^{*}P_{s3-W}(f+\alpha )+P_{s2-W}{\left(f\right)}^{*}P_{s1-W}(f+\alpha )\right\} & \mathrm{if}\;\alpha =m^{\prime }f_{s}\;-\;f_{s}^{\left(r\right)}\\ \underset{N\to \infty }{\lim}\frac{1}{W}E\left\{P_{s3-W}{\left(f\right)}^{*}P_{s2-W}(f+\alpha )\right\} & \mathrm{if}\;\alpha =m^{\prime }f_{s}\;+\;2f_{s}^{\left(r\right)}\\ \underset{N\to \infty }{\lim}\frac{1}{W}E\left\{P_{s2-W}{\left(f\right)}^{*}P_{s3-W}(f+\alpha )\right\} & \mathrm{if}\;\alpha =m^{\prime }f_{s}\;-2f_{s}^{\left(r\right)}\\ 0 & \mathrm{other}\;\alpha . \end{array}\right.$$

The peaks in SC_s_ appear grouped at point SC_s_($m^{\prime }f_{s}$, $f_{m}\pm mf_{s}$) and SC_s_($\pm k^{\prime }f_{s}^{\left(r\right)}\pm m^{\prime }f_{s}$, $f_{m}\pm f_{s}^{\left(r\right)}\pm mf_{s}$), where *m and m*′ are natural numbers, *k*′ is equal to 1 or 2, and the other positions yield a value of zero. Considering the harmonics of the sun gear rotating frequency, the peaks appear at frequencies $f_{m}\;\pm mf_{s}\;\pm kf_{s}^{\left(\;r\;\right)}$ in the Fourier spectrum. Thus, the peaks in ${\mathrm{SC}}_{s}(\alpha ,f)$ appear grouped at points SC_s_($m^{\prime }f_{s}$, $f_{m}\pm mf_{s}$) and SC_s_($\pm k^{\prime }f_{s}^{\left(r\right)}\pm m^{\prime }f_{s}$, $f_{m}\;\pm kf_{s}^{\left(r\right)}\;\pm mf_{s}$), where *k*, *k*′, *m*, and *m*′ are natural numbers.

### 4.3. Planet Gear Fault Case

The planet gear meshes with both the sun gear and the ring gear. The length of the transmission path from the fault meshing points to the accelerometer varies with the revolution of the planet gear. Therefore, the AM effect of the transmission path can be represented via a Hanning window with the planet carrier rotating frequency [5]. The localized fault vibration signal of the planet gear can be expressed as follows:

(17) $$x_{p}(t)=\left[1-\cos\left(2\pi f_{c}^{\left(r\right)}t\right)\right]\cdot \left[1-A\cos\left(2\pi f_{p}t+\phi \right)\right]\cdot \cos\left[2\pi f_{m}t+B\sin\left(2\pi f_{p}t+\varphi \right)+\theta \right]$$

where $f_{p}$ is the fault characteristic frequency of the planet gear, and $f_{c}^{\left(r\right)}$ is the rotating frequency of the planet carrier. The corresponding Fourier spectrum can be derived as follows:

(18) $$\left\{\begin{array}{c} X_{p}(f)=P_{p1}(f)+P_{p2}(f)+P_{p3}(f)\\ P_{p1}(f)=\sum_{m=-\infty }^{\infty }\delta \left[f-\left(f_{m}+mf_{p}\right)\right]\exp[j(m\varphi +\theta )]\left[J_{m}(B)+\frac{A}{2}J_{m-1}(B)\exp(j\phi )+\frac{A}{2}J_{m+1}(B)\exp(-j\phi )\right]\\ P_{p2}(f)=-\frac{1}{2}\sum_{m=-\infty }^{\infty }\delta \left[f-\left(f_{m}+mf_{p}+f_{c}^{\left(r\right)}\right)\right]\exp[j(m\varphi +\theta )]\left[J_{m}(B)+\frac{A}{2}J_{m-1}(B)\exp(j\phi )+\frac{A}{2}J_{m+1}(B)\exp(-j\phi )\right]\\ P_{p3}(f)=-\frac{1}{2}\sum_{m=-\infty }^{\infty }\delta \left[f-\left(f_{m}+mf_{p}-f_{c}^{\left(r\right)}\right)\right]\exp[j(m\varphi +\theta )]\left[J_{m}(B)+\frac{A}{2}J_{m-1}(B)\exp(j\phi )+\frac{A}{2}J_{m+1}(B)\exp(-j\phi )\right]. \end{array}\right.$$

It can be simplified as follows:

(19) $$X_{p}(f)=\left\{\begin{array}{ll} P_{p1}(f) & \mathrm{if}\;f=f_{m}+mf_{p}\\ P_{p2}(f) & \mathrm{if}\;f=f_{m}+mf_{p}+f_{c}^{\left(r\right)}\\ P_{p3}(f) & \mathrm{if}\;f=f_{m}+mf_{p}-f_{c}^{\left(r\right)}\\ 0 & \mathrm{other}\;f. \end{array}\right.$$

Thereby, the spectral peaks of the planet gear fault signal appear at frequencies $f_{m}\;\pm \;mf_{p}$ and $f_{m}\;\pm \;mf_{p}\;\pm \;f_{c}^{\left(\;r\;\right)}$. Considering the harmonics of the planet carrier rotating frequency, the peaks are present at frequencies $f_{m}\;\pm \;mf_{p}\;\pm \;kf_{c}^{\left(\;r\;\right)}$. The ${\mathrm{SC}}_{p}(\alpha ,f)$ of $x_{p}(t)$ can be deduced as follows:

(20) $${\mathrm{SC}}_{p}(\alpha ,f)\;=\left\{\begin{array}{ll} \underset{N\to \infty }{\lim}\frac{1}{W}E\left\{P_{p1-W}{\left(f\right)}^{*}P_{p1-W}(f+\alpha )+P_{p2-W}{\left(f\right)}^{*}P_{p2-W}(f+\alpha )+P_{p3-W}{\left(f\right)}^{*}P_{p3-W}(f+\alpha )\right\} & \mathrm{if}\;\alpha =m^{\prime }f_{p}\\ \underset{N\to \infty }{\lim}\frac{1}{W}E\left\{P_{p1-W}{\left(f\right)}^{*}P_{p2-W}(f+\alpha )+P_{p3-W}{\left(f\right)}^{*}P_{p1-W}(f+\alpha )\right\} & \mathrm{if}\;\alpha =m^{\prime }f_{p}\;+\;f_{c}^{\left(r\right)}\\ \underset{N\to \infty }{\lim}\frac{1}{W}E\left\{P_{p1-W}{\left(f\right)}^{*}P_{p3-W}(f+\alpha )+P_{p2-W}{\left(f\right)}^{*}P_{p1-W}(f+\alpha )\right\} & \mathrm{if}\;\alpha =m^{\prime }f_{p}\;-\;f_{c}^{\left(r\right)}\\ \underset{N\to \infty }{\lim}\frac{1}{W}E\left\{P_{p3-W}{\left(f\right)}^{*}P_{p2-W}(f+\alpha )\right\} & \mathrm{if}\;\alpha =m^{\prime }f_{p}\;+\;2f_{c}^{\left(r\right)}\\ \underset{N\to \infty }{\lim}\frac{1}{W}E\left\{P_{p2-W}{\left(f\right)}^{*}P_{p3-W}(f+\alpha )\right\} & \mathrm{if}\;\alpha =m^{\prime }f_{p}\;-2f_{c}^{\left(r\right)}\\ 0 & \mathrm{other}\;\alpha . \end{array}\right.$$

Equation (20) shows that the peaks in SC_p_ appear at SC_p_($m^{\prime }f_{p}$, $f_{m}\;\pm \;mf_{p}$) and SC_p_($\pm \;k^{\prime }f_{c}^{\left(r\right)}\;\pm \;m^{\prime }f_{p}$, $f_{m}\;\pm \;f_{c}^{\left(r\right)}\;\pm \;mf_{p}$), where *m and m*′ are natural numbers, and *k*′ is equal to 1 or 2. Considering the harmonics of the planet carrier rotating frequency, the peaks in SC_p_ appear grouped at point SC_p_($m^{\prime }f_{p}$, $f_{m}\;\pm \;mf_{p}$) and SC_p_($\pm \;k^{\prime }f_{c}^{\left(r\right)}\;\pm \;m^{\prime }f_{p}$, $f_{m}\;\pm \;kf_{c}^{\left(r\right)}\;\pm \;mf_{p}$), where *k*, *k*′, *m*, and *m*′ are natural numbers, and the other positions yield a value of zero.

In practical fault diagnosis, we focus on the spectral bands near the gear meshing frequency in SC or SCoh, and the fault-related peaks can be cross-verified through their spectral frequencies. Fault features typically appear as clustered patterns along both the spectral and cyclic frequency axes. Generally, a higher number of fault peaks with larger amplitudes corresponds to stronger fault evidence.

## 5. Simulation Analysis

This section validates the demodulation features derived for planetary gearbox diagnosis through a simulated signal analysis. The simulated signal for the sun gear fault is generated by Equation (13), and the main parameter values are shown in Table 1. Gaussian white noise is added to the simulated signal, resulting in a final signal-to-noise ratio of −15 dB. The signal duration is 20 s, and the sampling frequency is 2 kHz.

**Figure 2:**
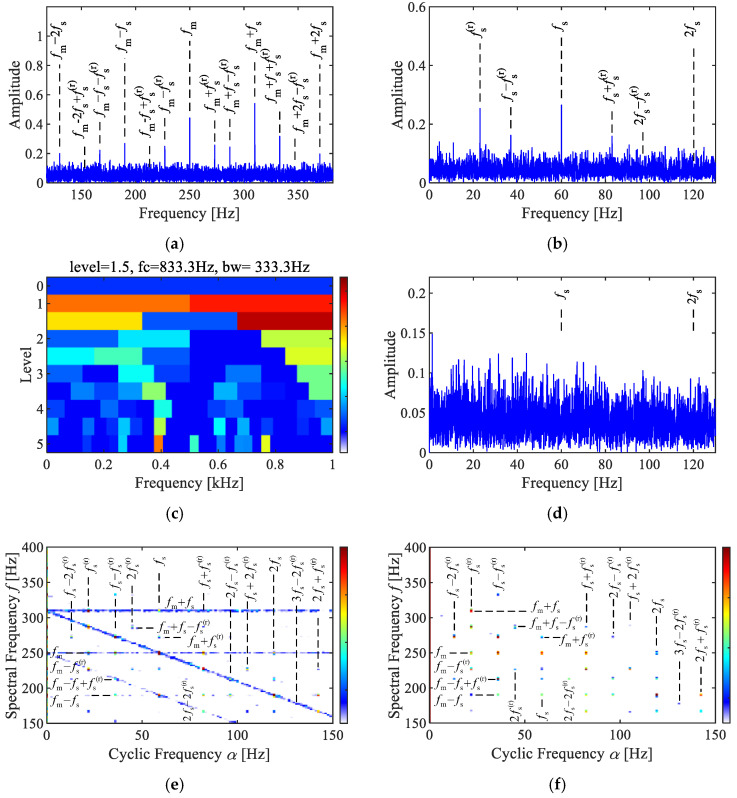
Analysis of the simulated signal. (**a**) Fourier spectrum, (**b**) envelope spectrum, (**c**) Fast-SK, (**d**) envelope spectrum via Fast-SK, (**e**) SC, (**f**) SCoh.

To validate the performance of the SC methods, a comparative analysis was conducted between SC, SCoh, the Fourier spectrum, the envelope spectrum, and the Fast Spectral Kurtosis (Fast-SK). The analysis results of the synthesized signal are illustrated in Figure 2.

The Fourier spectrum in Figure 2a shows features consistent with theoretical expectations, including sidebands related to the fault characteristics $f_{m}\;\pm f_{s}$ near the meshing frequency $f_{m}$, as well as composite sidebands due to the time-varying transmission paths $f_{m}\;\pm f_{s}\;\pm \;f_{s}^{\left(r\right)}$. The envelope spectrum in Figure 2b reveals modulation-related frequencies, such as $f_{s}$, $f_{s}^{\left(r\right)}$, $f_{s}\;\pm \;f_{s}^{\left(r\right)}$, and $2\;f_{s}\;-\;f_{s}^{\left(r\right)}$. After envelope demodulation, the modulation information is directly displayed, while the carrier information is removed.

The results of the Fast-SK analysis are presented in Figure 2c,d. The Fast-SK indicated that the optimal analysis frequency band is [667, 1000] Hz. However, no effective fault characteristic peaks were observed in the corresponding envelope spectrum. This signifies that the frequency bands associated with AM–FM characteristics can not be accurately detected by the Fast-SK, which aimed at identifying impulsive features. Thus, selecting an appropriate signal model is crucial for diagnosing faults in planetary gearboxes.

The results of the SC and SCoh analyses are shown in Figure 2e,f, both exhibiting a discrete point group. The characteristic peak points are located at cyclic frequency axes corresponding to the sun gear fault frequency $(1,2)f_{s}$, rotating frequency $(1,2)f_{s}^{\left(r\right)}$, and their combination frequencies, including $f_{s}\;\pm \;(1,2)f_{s}^{\left(r\right)}$, $2f_{s}\;\pm \;(1,2)f_{s}^{\left(r\right)}$, and $3f_{s}\;-\;2f_{s}^{\left(r\right)}$. The characteristics in spectral frequency axis corresponded to features around the meshing frequency, such as $f_{m}$, $f_{m}\;\pm f_{s}$, $f_{m}\;\pm [f_{s}\;-\;f_{s}^{\left(r\right)}]$. These are consistent with the derived theoretical characteristics in Equation (16). The difference between SC and SCoh is that SC reflects the energy characteristics of modulation features within the signal. As illustrated in Figure 2e, prominent peaks at 250 Hz and 310 Hz result in two sets of linear features in SC. In contrast, SCoh is not influenced by local energy values and instead displays the relative amplitude of the modulation features, resulting in all features appearing as distinct points.

## 6. Experimental Validation

To verify the derived features for planetary gearboxes, we conducted a comparative analysis of SC and traditional envelope spectrum demodulation through fault experiments on a planetary gearbox test bench.

### 6.1. Test Setting

**Figure 3:**
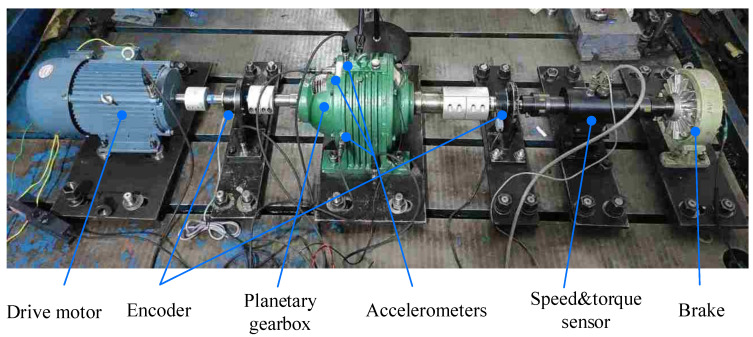
Planetary gearbox transmission test rig.

**Figure 4:**
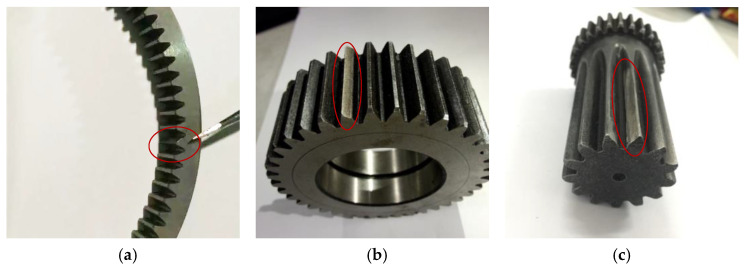
Locally faulty gears. (**a**) Ring gear, (**b**) sun gear, (**c**) planet gear, the circles indicate the faulty teeth.

The planetary gearbox test bench is illustrated in Figure 3. The power was inputted from the motor to the sun gear, transmitted through the gear train, and outputted via the planet carrier to a magnetic powder brake. The accelerometer was vertically fixed on the planetary gearbox housing using magnetic bases. To simulate local tooth spalling faults, a portion of the tooth surface was artificially removed from one tooth of the ring gear, sun gear, and planet gear, respectively, with maximum depths of 1 mm and widths of about 3 mm. The faults on the sun gear and ring gear were located on the meshing surfaces, and the planet gear fault was on the tooth surface that meshed with the ring gear. The faulty gears are illustrated in Figure 4. In the tests, the three faulty gears were individually integrated into the planetary gearbox one by one. The tests configurations were maintained at an input speed of 23.38 Hz under a load torque of about 20 Nm. Table 2 lists the geometric parameters characterizing the planetary gearbox. Table 3 lists the rotating frequencies of all components and their corresponding fault characteristic frequencies with localized gear faults.

### 6.2. Ring Gear Fault Case

**Figure 5:**
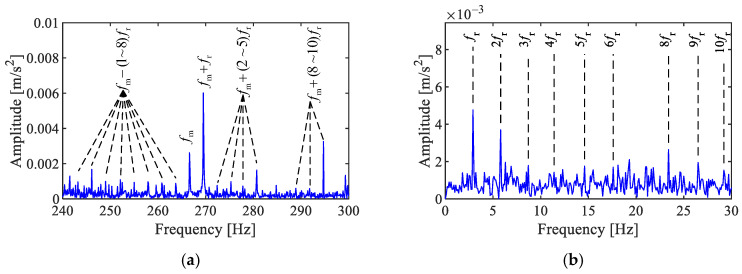

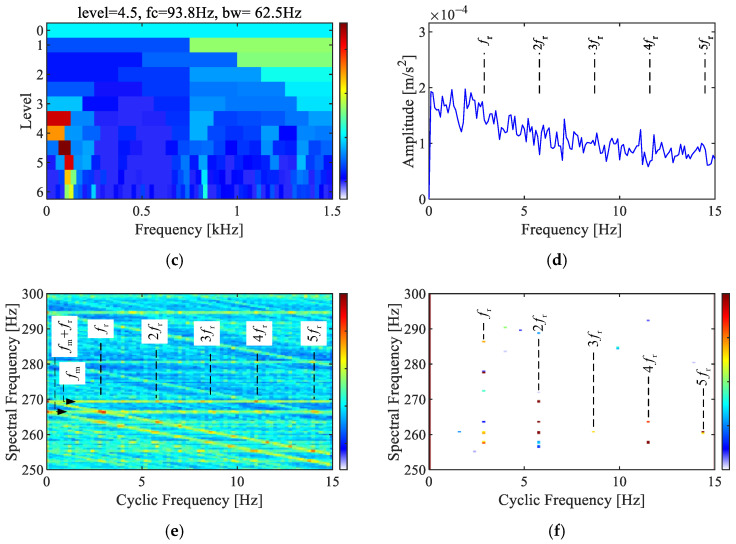
Analysis of the ring gear fault signal. (**a**) Fourier spectrum, (**b**) envelope spectrum, (**c**) Fast-SK, (**d**) envelope spectrum via Fast-SK, (**e**) SC, (**f**) SCoh.

The analysis results of the synthesized signal are illustrated in Figure 5. As shown in Figure 5a, the spectrum within the first meshing frequency band reveals the peaks of the meshing frequency and modulated sidebands from the multi-order of the fault characteristic frequencies of the ring gear $f_{m}\;-\;(1\sim 8)\;f_{r}$ and $f_{m}\;+\;(1\sim 5,8\sim 10)\;f_{r}$. The prominent peaks of the envelope spectrum in Figure 5b were observed at the fault characteristic frequency of the ring gear and its harmonics $\;(1\sim 6,8\sim 10)\;f_{r}$. These align with the typical spectral and demodulated characteristics of AM–FM signals.

The results of fault feature extraction using Fast-SK are shown in Figure 5c,d, with the optimal analysis band identified as [62.6, 125] Hz. The obtained envelope spectrum had low amplitude, with no characteristic peak associated with the planetary gearbox. It is evident that the optimal band obtained from the impact extraction method contains few meshing characteristics.

In the SC displayed in Figure 5e, a cluster of discrete peaks are present in the frequency range of 250 to 270 Hz, with cyclic frequencies corresponding to the 1st to 5th harmonics of the ring gear fault characteristic frequency $\;(1\sim 5)\;f_{r}$. The two sets of features at spectral frequencies of 266 Hz and 269 Hz correspond to the meshing frequency $f_{m}$ and the ring gear fault sideband $f_{m}\;+\;f_{r}$, respectively. The horizontal and vertical interval points correspond to the ring gear fault characteristic frequency $f_{r}$. In the SCoh, these clustered fault characteristic peaks become more distinct after energy normalization. The phenomenon of SC and SCoh is consistent with the theoretical conclusions and reveals the presence of a localized fault in the gear ring.

### 6.3. Sun Gear Fault Case

**Figure 6:**
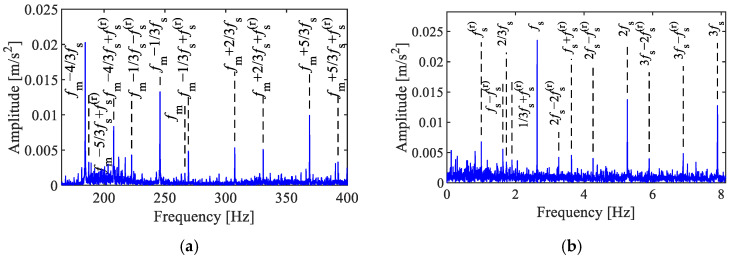

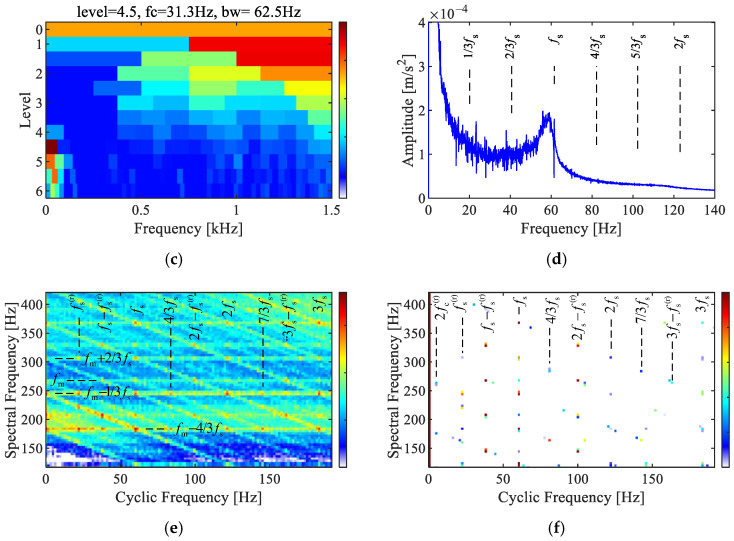
Analysis of the sun gear fault signal. (**a**) Fourier spectrum, (**b**) envelope spectrum, (**c**) Fast-SK, (**d**) envelope spectrum via Fast-SK, (**e**) SC, (**f**) SCoh.

The results of the sun gear fault signal analysis are displayed in Figure 6. Due to the additional modulation effects of the time-varying transmission path, the characteristics of the sun gear fault are more complex. As illustrated in Figure 1a, sidebands corresponding to the sun gear fault characteristics $f_{m}\;+\;(-4,-1,2,5)f_{s}\;/\;3$ and their combination frequencies with the sun gear rotating frequency $f_{m}\;+\;(-4,-1,2,5)f_{s}\;/\;3+\;f_{s}^{\left(r\right)}$ are prominent in the Fourier spectrum, spaced at intervals of the fault characteristic frequency $f_{s}$. The envelope spectrum shows significant peaks of the sun gear fault and its harmonics $(1,2,3)f_{s}$, as well as the combination frequencies with the sun gear rotating frequencies $(1,2,3)f_{s}\;-\;f_{s}^{\left(r\right)}$ and $3f_{s}\;-\;2\;f_{s}^{\left(r\right)}$

As shown in Figure 6c,d, the Fast-FK indicates that the optimal analysis frequency band is [0, 62.5] Hz. The characteristics in the envelope spectrum after low-pass filtering are concentrated below 70 Hz, with a subtle peak identified at the sun gear fault frequency.

As shown in Figure 6e,f, clear sun gear fault characteristic frequencies $(1,4\;/\;3,2,7\;/\;3,3)f_{s}$ and their combination frequencies with the sun gear rotating frequency $(1,2,3)f_{s}\;-\;f_{s}^{\left(r\right)}$ are present between [150, 400] Hz of both the SC and SCoh. The spectral frequencies of the feature points are denoted as the meshing frequency $f_{m}$, and the combined frequency with the sun gear fault characteristic frequency $f_{m}\;-\;4\;/\;3f_{s}\;$, $f_{m}\;-\;1\;/\;3f_{s}\;$, $f_{m}\;+\;2\;/\;3f_{s}\;$, etc., spaced at intervals of $1\;/\;3f_{s}$ or $f_{s}$, which are similar to the characteristic frequencies shown in Figure 6a. The analysis results indicate that localized damage occurred on the sun gear.

### 6.4. Planet Gear Fault Case

**Figure 7:**
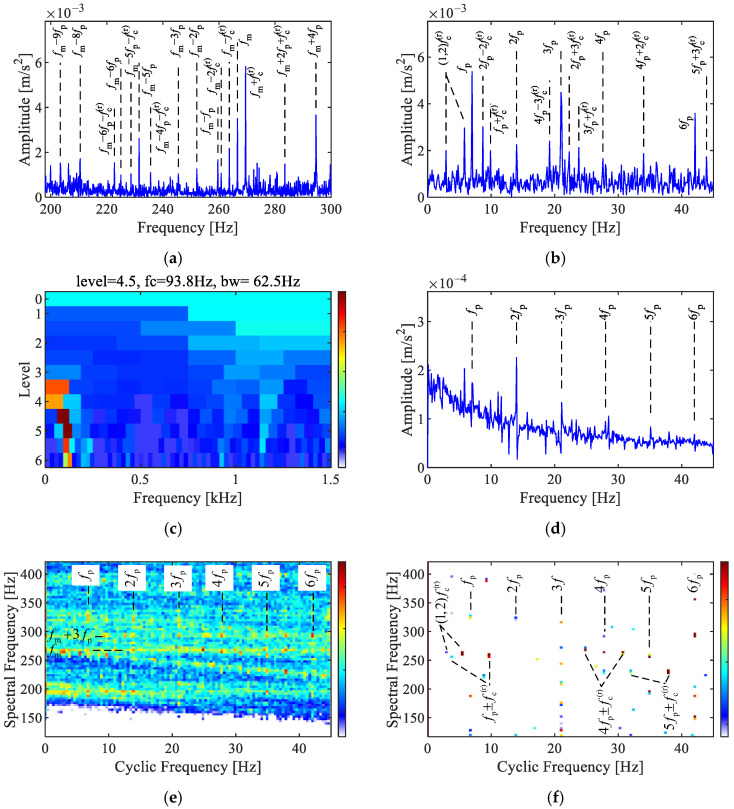
Analysis of the planet gear fault signal. (**a**) Fourier spectrum, (**b**) envelope spectrum, (**c**) Fast-SK, (**d**) envelope spectrum via Fast-SK, (**e**) SC, (**f**) SCoh.

The results of the planet gear fault signal analysis are shown in Figure 7. The Fourier spectrum in Figure 7a shows multiple sidebands modulated by the planet carrier rotating frequency $f_{m}\;+\;(-2,-1,1)\;f_{c}^{\left(r\right)}$, the first to ninth order sidebands of the planet gear fault characteristic frequencies $f_{m}\;\;-\;(1\sim 9)\;f_{p}$, $f_{m}\;\;+\;4\;f_{p}$, and combination sidebands $f_{m}\;\;+2\;f_{p}\;+\;f_{c}^{\left(r\right)}$, and $f_{m}\;\;-\;(4\sim 6)\;f_{p}\;-\;f_{c}^{\left(r\right)}$. As shown in Figure 7b, the dominant peaks in the envelope spectrum are the fault characteristic frequencies of the planet gear and its harmonics $\;(1\sim 4,6)\;f_{p}\;$, along with combination frequencies related to the rotating frequencies of the planet carrier $2\;f_{p}\;-\;2f_{c}^{\left(r\right)}$, $f_{p}\;+\;f_{c}^{\left(r\right)}$. They are consistent with characteristics of the planetary gear fault signal model.

The results via Fast-SK are shown in Figure 7c,d. The envelope spectrum of the optimal analysis band [62.6, 125] Hz reveals the first to sixth orders of the fault characteristic frequency $\;(1\sim 6)\;f_{p}$.

As displayed in Figure 7e,f, both the SC and SCoh revealed the first to sixth orders of the planet gear fault characteristic frequencies $\;(1\sim 6)\;f_{p}$ in the cyclic frequency axis. Two distinct spectral frequencies in the SC correspond to the meshing frequency $f_{m}$, and the combined frequency with the planet gear fault characteristic frequency $f_{m}\;+\;3\;f_{p}\;$. Compared to SC, SCoh exhibits less noise interference, and combination frequencies with the rotating frequencies of the planet carrier $(1,4,5)f_{p}\;\pm \;f_{c}^{\left(r\right)}$ are clearly revealed in SCoh. These characteristics indicate the fault in the planet gear of the tested planetary gearbox.

### 6.5. Discussion

The SC features from three experimental data align with theoretical conclusions of this paper, successfully uncovering local fault characteristics of three types of gears. The grouped SC feature points indicate that the fault signals of the planetary gearbox predominantly exhibit AM–FM characteristics rather than impulsive response characteristics. The Fast-SK results display distinct fault features only in the case of planetary gear faults, further supporting this observation. The derived fault feature expressions provide theoretical guidance for SC-based fault diagnoses of planetary gearboxes. The SC analysis can be conducted directly within the meshing area, circumventing the challenges of determining resonance frequencies inherent in impulse response models. Since the theoretical features appear at specific frequencies in both the horizontal and vertical coordinates, cross-validation between the two coordinates can enhance the precision of fault feature source localization, which is a major advantage of the demodulation analysis of AM–FM signals based on the SC method.

## 7. Conclusions

Planetary gearbox vibration signals have a multiple-modulation nature, and their Fourier spectra show complex sideband structures. This poses a challenge to fault feature extraction via sideband analysis. This paper aimed to establish an SC-based theory for a planetary gearbox fault diagnosis. The theoretical demodulation characteristics of AM–FM signals are derived in frequency domains, resulting in grouped discrete characteristic points in the two-dimensional spectrum, with their cyclic frequencies equal to the modulation frequencies, and their spectral frequencies equal to the combination of the carrier and modulation frequencies. This differs significantly from the vertical line characteristics exhibited by impulse signals, as the accurate spectral frequencies can precisely identify the source of the modulation characteristics. Further, the spectral correlation features of planetary gearbox fault vibration signals are derived in closed form to facilitate fault diagnoses, including the fault conditions of the ring gear, sun gear, and planet gear. The SC can offer more profound and accurate demodulation information of planetary gearbox faults than the traditional envelope demodulation method. The methods and derived conclusions are validated through simulation analysis and experiments, and localized faults on the ring, sun, and planet gears are successfully diagnosed.

## Figures and Tables

**Table 1:** Simulated signal parameters.

Parameters	Values
Sun gear rotating frequency $f_{s}^{\left(r\right)}$	23 Hz
Sun gear fault frequency $f_{s}\;$	60 Hz
Meshing frequency $f_{m}$	250 Hz
AM intensity *A*	1.9
FM intensity *B*	0.6
Initial phases of AM ϕ	5.7
Initial phases of FM φ	3.1
Initial phases of meshing θ	4.9

**Table 2:** Configuration parameters of gearboxes.

Gear	Sun	Planet (Number)	Ring
Gear teeth	13	38 (3)	92

**Table 3:** Characteristic frequencies of planetary gearboxes.

Feature	Frequency (Hz)	Feature	Frequency (Hz)
Sun gear rotating $f_{s}^{\left(r\right)}$	23.38	Sun gear fault $f_{s}$	61.45
Ring gear rotating $f_{r}^{\left(r\right)}$	0	Ring gear fault $f_{r}$	2.89
Planet gear rotating $f_{p}^{\left(r\right)}$	4.11	$\mathrm{Planet}\;\mathrm{gear}\;\mathrm{fault}\;f_{p}$	7.01
Planet carrier rotating $f_{c}^{\left(r\right)}$	2.89	$\mathrm{Meshing}\;f_{m}$	266.29

## Data Availability

The raw data supporting the conclusions of this article will be made available by the authors on request.
